# Supplementary material for: Emitting long-distance spiral airborne sound using low-profile planar acoustic antenna
Source: Nat Commun. 2021 Mar 31;12:2006. doi: 10.1038/s41467-021-22325-7 (PMC8012347; doi:10.1038/s41467-021-22325-7)
Supplement: Supplementary file 1 — Supplementary Information [file 41467_2021_22325_MOESM1_ESM.pdf]

Supplementary Information for
Emitting long-distance spiral airborne sound using low-profile planar acoustic antenna

Shuxiang Gao,[1] Yunbo Li,[2] Chengrong Ma,[1] Ying Cheng,[1,*] and Xiaojun Liu[1,†]

[1]*Key Laboratory of Modern Acoustics, Department of Physics and Collaborative Innovation Center of Advanced Microstructures, Nanjing University, Nanjing 210093, China*

[2]*State Key Laboratory of Millimeter Waves, Southeast University, Nanjing 210096, China*

(Dated: February 1, 2021)

* chengying@nju.edu.cn

† liuxiaojun@nju.edu.cn

Note 1. SAW IMPEDANCE

Considering a uniform plane wave incident at angle θ_1 onto the plane interface between two mediums with refraction index of n_1 and n_2, a refracted wave will be yielded into the medium 2 at angle θ_2 with the normal (see Supplementary Fig. 1). Using the Snell's Law of refraction, we can obtain a relation between the angle of incidence θ_1 and the angle of refraction θ_2 [1]:

$$\frac{\sin\theta_2}{\sin\theta_1} = \frac{k_1}{k_2} = \frac{n_1}{n_2}. \tag{1}$$

We define the characteristic impedance referred to the z direction as the spoof surface acoustic wave (SAW) impedance, assuming that there is no returning wave in both sides. Thus, the SAW impedance for medium 1 is

$$Z_1 = Z_i \cos\theta_1, \tag{2}$$

in which Z_i is the characteristic impedance of the incident side medium, while the load impedance Z_2 in medium 2 is the SAW impedance for the refracted wave:

$$Z_s = Z_2 = Z_0 \cos\theta_2 = Z_0\sqrt{1-\left(\frac{n_1}{n_2}\sin\theta_1\right)^2}. \tag{3}$$

For the present case, the medium 1 is the designed metasurface with the calculated refractive index $n_1 = n$ and the medium 2 is air with $n_2 = 1$, $Z_0 = \rho_0 c_0$. Considering the angle of incidence θ_1 is 90 °(actually acoustic surface waves are excited by an air-side source and in essence it is the case of total reflection), the SAW impedance can be reduced to

$$Z_s = Z_0\sqrt{1-n^2}. \tag{4}$$

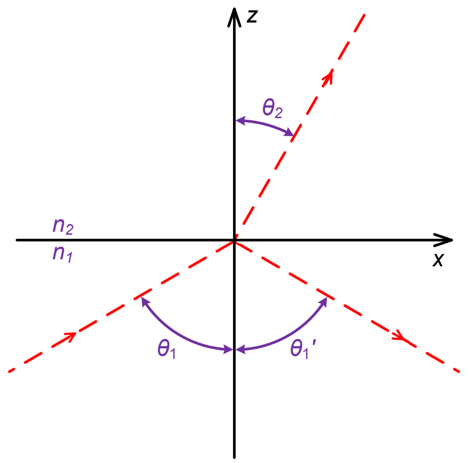

Supplementary Fig. 1. Incidence onto the interface between two different mediums at a certain angle θ_1.

Note 2. WAVE VECTOR TRANSFER

The formation of holographic impedance acoustic antenna is based on two fundamental theories: holographic theory and artificial impedance metasurface theory. The function achieved by the artificial impedance metasurface is to modulate the SAW mode, and its principle is the transfer of the wave vector. As shown in Supplementary Fig. 2, assuming that the units in a period serve as a whole, by converting the wavefront of incident surface acoustic wave into the wavefront of radiation waves with regards to appropriate parameters, the near-field surface wave could be emitted to the far field (i.e., shift the wave vector from point I to II). Similarly, when a certain pattern of sound waves is not willing to be radiated, we can also restrict it to the near field through appropriate wave vector transfer (i.e., transfer the wave vector from point I to III).

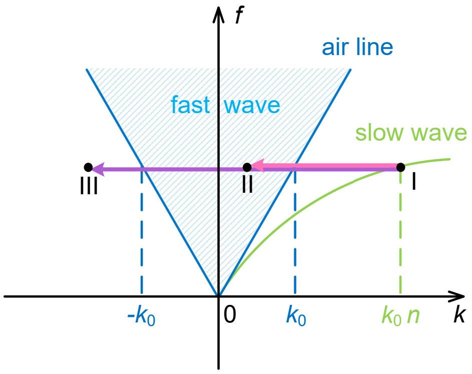

Supplementary Fig. 2. Schematic diagram of transferring an evanescent surface wave (slow wave) into a radiation wave (fast wave). The green solid line and blue solid line indicate the dispersion curve propagating along the surface and in the air, respectively.

Note 3. ANTENNA SIZE

We would like to note that for the modulated acoustic antenna, the larger the aperture is, the better the antenna outgoing effect can be achieved. Since the surface wave is periodically modulated, the radius of the antenna should be at least more than one period. Therefore, we show the simulation results of the total transverse size of only 3 wavelengths (18 cm) in Supplementary Fig. 3. The wavefront phase and normalized amplitude distributions are consistent with that in Fig. 4d of the main text and the critical distance turns out to be 0.14 m here. After only 1.5 modulation periods, the vortex phase can still be obtained in the far field, while the amplitude is fairly small because the modulated emission energy from the surface wave conducting on the metasurface is quite limited.

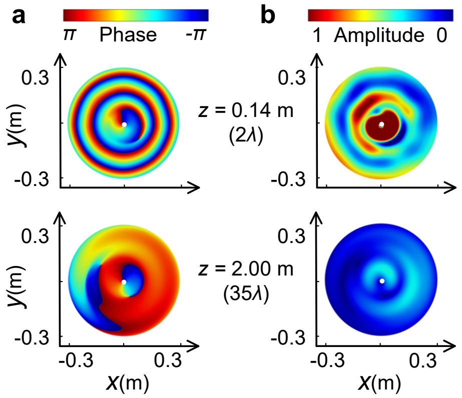

Supplementary Fig. 3. Cross-sectional views of simulated sound (a) phase and (b) amplitude distribution with structure radius of 1.5 modulation periods, located at 0.14 m and 2.00 m, respectively.

Note 4. ACOUSTIC VORTICES

Supplementary Fig. 4 displays the experimentally measured phase diagrams of sound pressure field within about one period. The measurements are taken in the remote area along the z direction at intervals of one centimeter. It can be seen that the phases vary gradually in the x-y plane and this variation rotates uniformly around the center axis in one cycle, which clearly demonstrates the characteristics of the first-order spiral field. Note that the amplitude diagrams of sound pressure field in the remote area remains almost constant as shown in Fig. 4d of the main text, so they are not described repetitively here.

In order to rule out the interference of other factors, we also give the background sound phase and pressure amplitude without the metasurface in Supplementary Fig. 5. Measurements are taken at two typical locations in the direction of propagation, one at the critical distance 1.57 m and the other at 2.00 m in the far field. Since there is only

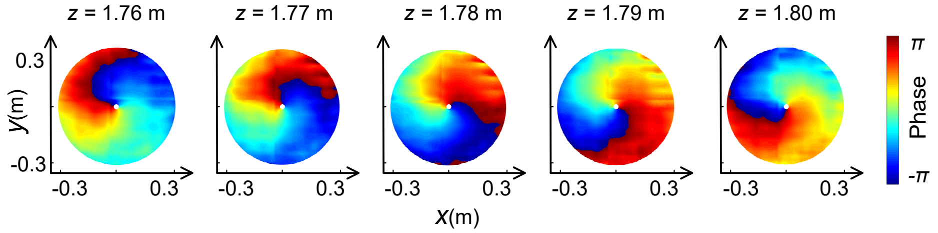

Supplementary Fig. 4. Cross-sectional views of experimentally measured phase diagrams of spiral sound within one period from 1.76 m to 1.80 m.

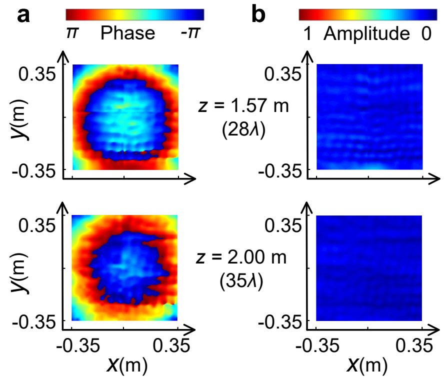

Supplementary Fig. 5. Cross-sectional views of experimentally measured (a) phase and (b) amplitude distribution of background sound pressure field located at 1.57 m and 2.00 m, respectively.

one point-like source, the phase of the background field sound pressure presents a concentric circle distribution as shown in Supplementary Fig. 5a. For the corresponding normalized amplitude of the background field, as shown in Supplementary Fig. 5b, due to the inevitable wave diffraction, the far-field sound pressure is so weak that the amplitude approaches zero, which further confirms the function of our metasurface to convert the surface evanescent wave into the collimated sound vortex beam.

Note 5. NEW PHYSICS WITH APPEALING APPLICATIONS

Indeed, the topic of acoustic OAM generation has been widely exploited. However, we would like to emphasize that our strategy offers the possibility for the first time to produce long-distance spiral airborne sound using low-profile planar acoustic antenna. Please note three important differences between the active/passive designs in previous literatures and our proposal in the manuscript.

(1) Active designs are essentially based on acoustic phased array technique that uses multiple independently regulated acoustic transducers to form the array, producing reconfigurable spatial phase distribution of the desired spiral shape. However, in order to pursue a nearly continuous spiral wavefront for long-distance self-collimated propagation, it is often necessary to depend on a huge number of transducers forming an extremely large-scale acoustic array, and to operate individual units independently through complicated electric system composed of stimulant signal generation and power amplification, ensuring that the phase of the drive signal corresponding to each transducer is precisely modulated and powerful enough. Therefore, the active generation method of acoustic OAM requires large space occupation and complicated circuit, and each transducer unit itself has a certain size, which also brings difficulties to its application in the high-frequency range.

On the contrary, the metasurface we proposed relies on passive technique that overcomes the inherent limitations of complex circuit control associated with the structure, but instead creates a spiral phase distribution required for the vortex field by using an impedance metasurface with a discontinuous impedance gradient, which has the advantages of flat structure and relatively lower cost. Moreover, the fabricated metasurface in the manuscript is composed of 7660 elements, with the deep-subwavelength element period of d = 6 mm (that is, about $\lambda/10$, far smaller than the

operating wavelength of about 56.7 mm at 6 kHz).

(2) Furthermore, please note that the proposed method is also significantly superior in passive methods. Firstly, our metasurface does not depend on resonances produced by elements with composite internal micro-structure. Secondly, the acoustic diffraction effects require that the dimensions of the structure in the thickness direction should be greater than or equivalent to the acoustic wavelength, while our structure is an unconstrained flat, ultra-thin structure. Although all current planar structures claimed to be thin themselves, we would like to note that the excitation sound sources of incident plane waves and the required propagation distance to avoid near-field effects of practical transducers inevitably take up bulky space in these transmissive configurations, as these designs are based on the modulation of transmitted propagating waves.

On the contrary, what we are focusing on is the modulation and conversion of evanescent spoof surface acoustic waves, which is a guided wave pattern that only propagates along the surface of the rigid periodic structure on the fluid side. We are deliberately investigating the holographic interferogram as a 2D modulated artificial acoustic impedance metasurface and the subsequent complex inter-modal interaction between cylindrical surface waves and the spatially-modulated impedance boundary condition. Such complex interplay cannot be traced in transmission-type metasurfaces mentioned above, indicating that our proposal is not a variant of the previous passive structures and they are based on very different physics principles. Moreover, our point excitation source can be positioned in the close vicinity of the metasurface, which functions as a coplanar structure as a whole, realizing a real low profile.

(3) In addition to the distinct physical mechanism, please note that due to its unique air-compatible conductivity, ultra-thin structure and ability to carry sub-wavelength information to far fields, this metasurface may lead to a wide range of extensive and significant applications. Different from other OAM generators, the air-compatible conductivity allows the sound wave to propagate to a remote distance in the far-field without resorting to the additional waveguides (please note that these waveguides are ordinarily employed in most current metasurface designs for generation and transmission of acoustic vortex, but not applicable or cumbersome in many practical scenarios), which should be promising in non-contact manipulation of particles, acoustic long-distance communication and non-intrusive detection of the energy flux, and the evolution of acoustic waves in the complex fluctuating system.

Additionally, although we mainly focused on the metasurface for the generation of long-distance spiral beam in the manuscript, the findings of impedance metasurface can enable flexible conversion of spoof surface acoustic waves for realizing versatile functional devices with superior performance. Thus, the proposed strategy provides a feasible approach to design integrated and small-sized system-level antenna as well as being loaded on other functional devices of irregular shape, which can be extended far beyond the scope of original results in the manuscript and utilized in acoustic multi-mode communication as a consequence of the flexible design of impedance surfaces and integration of different modes such as various angular momentums. For example, Supplementary Fig. 6 shows preliminary illustrations of acoustic dual-frequency OAM-dependent antenna, which can emit vortices with different charge-m at different frequencies simultaneously. Furthermore, Supplementary Fig. 7 illustrates the acoustic dual-angle OAM-dependent antenna, which radiate vortex beams along predesigned horizontal azimuth and pitch angles with different charge-m. These results clearly illustrate the broad application prospects in the field of signal multiplexing.

Supplementary References

[1] Simon Ramo, John R Whinnery, and Theodore Van Duzer. *Fields and waves in communication electronics.* John Wiley & Sons, 2008.

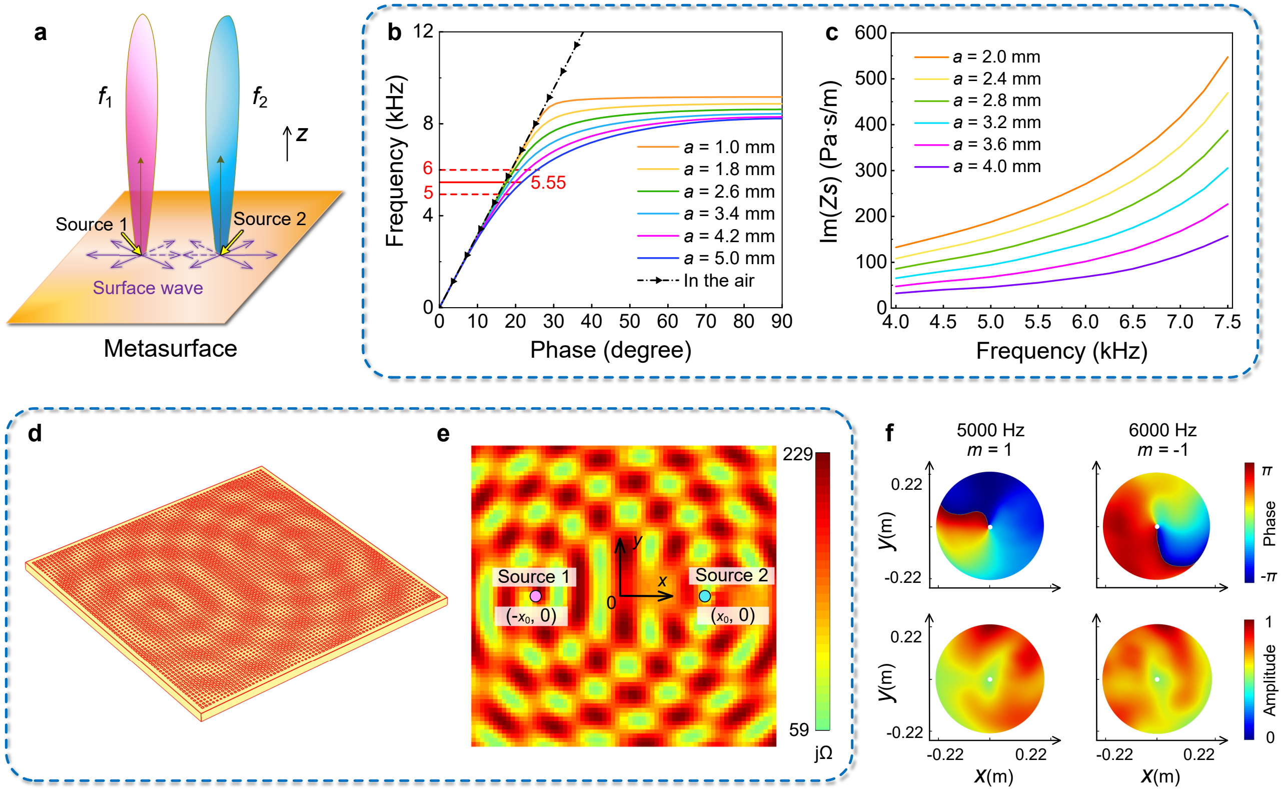

Supplementary Fig. 6. **Demonstration of dual-frequency OAM-dependent antenna.** (a) Schematic diagrams. The spoof SAW are launched by two monopolar sound sources with different frequencies of f_1 and f_2 (yellow pentagram) located adjacent to the metasurface antennas (orange plate), as illustrated by the divergent purple lines. These surface waves carrying zero OAM are scattered into the desired far-field wave beams with different artificial OAM m along z direction. (b) Dispersion curves of unit cells and (c) imaginary part of the surface impedance Z_s with different hole sizes a, while the black line with triangle marks denotes the air line. (d) The structure details and (e) impedance distribution of the designed square metasurface. (f) Simulated phase and amplitude distributions of the helical wavefronts at the observation planes located at 2.00 m away from the metasurface, respectively. The left panels indicate a vortex beam at $f_1 = 5000$ Hz with topological charges $m = 1$, while the right panels show the other vortex beam at $f_2 = 6000$ Hz with topological charges $m = -1$. The white dots refer to the geometric centers of the phase cross sections. See Supplementary Movie 3 for the dynamic view of dual-frequency OAM-dependent acoustic vortex beam.

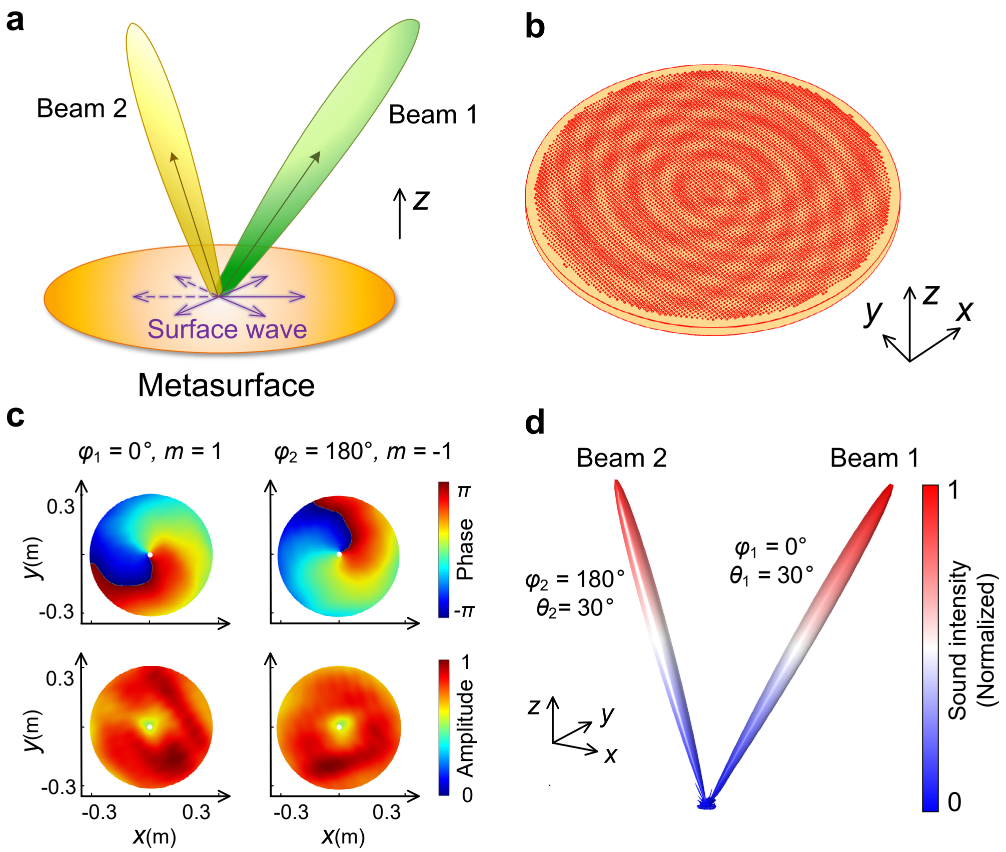

Supplementary Fig. 7. Demonstration of dual-angle OAM-dependent antenna. (a) Schematic diagrams. The spoof SAW are launched by a monopolar sound source (yellow pentagram) located adjacent to the metasurface antennas (orange plate), as illustrated by the divergent purple lines. These surface waves carrying zero OAM are scattered into the desired far-field wave beams along different horizontal azimuth ϕ_i and pitch angles θ_i. (b) The structure details of the designed circular metasurface. (c) Simulated phase and amplitude distributions of the helical wavefronts at the observation planes located at 2.00 m away from the metasurface, respectively. The left panels indicate a vortex beam along $\theta_1 = 30^\circ$ and $\phi_1 = 0^\circ$ with topological charges $m = 1$, while the right panels show the other vortex beam along $\theta_2 = 30^\circ$ and $\phi_2 = 180^\circ$ with topological charges $m = -1$. The white dots refer to the geometric centers of the phase cross sections. (d) Corresponding far-field radiation pattern. See Supplementary Movie 4 for the dynamic view of dual-angle OAM-dependent acoustic vortex beam.
