# Supplementary material for: Emitting long-distance spiral airborne sound using low-profile planar acoustic antenna
Source: Nat Commun. 2021 Mar 31;12:2006. doi: 10.1038/s41467-021-22325-7 (PMC8012347; doi:10.1038/s41467-021-22325-7)
Supplement: Supplementary file 3 — Description of Additional Supplementary Files [file 41467_2021_22325_MOESM3_ESM.pdf]

Description of Additional Supplementary Files

File Name: Supplementary Movie 1.

Description: The time evolution of first-order spiral vortex in the far field. Its snapshot is shown in Fig. 4b of the main text. For a particular moment, it is observed that the field pressure is separated into two poles like a ``Taiji'' diagram and the line on which the pressure is zero forms the shape of ``S''. The wavefront presents a total phase shift of 2π over each complete turn, which rotates like a neon light rotation.

File Name: Supplementary Movie 2.

Description: The wavefront distribution of the higher-order vortex beams with topological charges m = 2 to 4. One can see that the peak and valley areas of the pressure appear alternately around the central area. As the topological charge increases, the number of peaks and valleys also increases to twice of the topological charge, which is consistent with the theoretical predictions.

File Name: Supplementary Movie 3.

Description: The acoustic dual-frequency OAM-dependent antenna, which emit two vortices, among them one vortex beam at f_1 = 5000 Hz with topological charges m = 1, while the other vortex beam at f_2 = 6000 Hz with topological charges m = -1, as illustrated in Supplementary Fig. 6.

File Name: Supplementary Movie 4.

Description: The acoustic dual-angle OAM-dependent antenna, which emit two vortices, among them one vortex beam along θ_1 = 30° and φ_1 = 0° with topological charges m = 1, while the other vortex beam along θ_2 = 30 and φ_2 = 180° with topological charges m = -1, as illustrated in Supplementary Fig. 7.
